# Analysis of the Epidemiological Profile of Typhoid Fever in the State of Pará Between the Years 1999 and 2018

**DOI:** 10.1155/ijm/3747557

**Published:** 2025-11-06

**Authors:** Danielle Vieira Pina de Carvalho, Anderson Nonato do Rosario Marinho, Daniela Cristiane da Cruz Rocha

**Affiliations:** Instituto Evandro Chagas, Ananindeua, Pará, Brazil

**Keywords:** epidemiology, prevalence, *Salmonella* Typhi, typhoid fever

## Abstract

*Salmonella* Typhi is the causative agent of typhoid fever, a notifiable disease characterized by prolonged fever and gastrointestinal symptoms that may worsen and lead to death. This study was aimed at determining the epidemiological profile of typhoid fever based on clinical cases treated between 1999 and 2018 at the Evandro Chagas Institute, located in the state of Pará, Brazil. A total of 683 cases were analyzed, confirmed through PCR, stool culture, or blood culture. The findings revealed a consistent annual pattern in the number of reported cases, with the highest incidence occurring between June and November. The majority of affected individuals resided in the municipality of Belém, were male, had low levels of formal education, and were predominantly students. The age range of affected individuals varied from 1 to 88 years, with the majority of cases occurring in adults aged 20–59 years. Most cases occurred in urban areas with brick housing, biological septic tanks, dry soil, regular garbage collection, and access to public water supplies. Consumption of potentially contaminated foods, especially açaí, was identified as a probable source of infection. The most frequently reported symptoms included fever, headache, diarrhea, chills, abdominal pain, nausea, vomiting, myalgia, and asthenia. These findings highlight the need to expand knowledge about the still limited epidemiology of typhoid fever in this region in order to support the implementation of effective control and prevention strategies.

## 1. Introduction

The genus *Salmonella*, belonging to the Enterobacteriaceae family, comprises gram-negative, facultatively anaerobic, nonsporulating bacilli, most of which are motile via peritrichous flagella. The etiological agent of typhoid fever (TF) is *Salmonella enterica* subspecies *enterica*, serotype Typhi. Transmission occurs primarily through the ingestion of water and food contaminated with feces from infected individuals [1, 2].

According to the World Health Organization (WHO), there are an estimated 11–20 million new cases of TF globally each year, resulting in approximately 128,000–161,000 deaths [3, 4]. In Brazil, TF has been classified as a notifiable disease since 1961. The Brazilian Ministry of Health reports that the disease is endemic, often with overlapping outbreaks, and is closely associated with inadequate sanitation infrastructure and substandard individual and collective hygiene practices. TF is a major public health concern, particularly in the Amazon region. The state of Pará, for example, accounts for a substantial share of cases and frequent outbreaks. Between 2001 and 2019, 1362 cases were reported in Pará, representing 22.9% of all cases nationwide [5–7].

Despite its compulsory notification status due to its high epidemic potential, TF remains underreported. This underreporting is partly due to challenges in clinical recognition and laboratory confirmation, particularly the impact of prior antibiotic use, which can hinder the isolation and identification of *S.* Typhi [8, 9].

The primary factor contributing to the spread of TF is the lack of basic sanitation. Studies assessing the quality of sanitation services have shown that the North and Northeast regions of Brazil have historically recorded the poorest indicators. The state of Pará remains below the national average in terms of access to sanitation, with peripheral areas being especially vulnerable to waterborne diseases [7, 10–12].

The investigation of cases and outbreaks is essential for understanding the scope of the disease and for the development of preventive strategies. Epidemiological surveillance plays a key role in ensuring systematic monitoring and the effective implementation and evaluation of control measures [13, 14].

In the 21st century, TF continues to pose a serious global health challenge. Its persistence and spread require further in-depth investigation. Limited recognition of the disease is driven by multiple factors, including restricted access to medical care, a disproportionate burden on low-income communities that lack laboratory services, and difficulties in enacting public policies for the detection and monitoring of new cases. Additionally, the overuse of antibiotics, often readily available and used for empiric treatment, has contributed to the emergence of multidrug-resistant (MDR) strains, further complicating control efforts [15].

From this perspective, and with the aim of contributing to a more accurate understanding of the epidemiological profile of TF in the state of Pará, this study seeks to describe clinical cases treated at the Evandro Chagas Institute (IEC) between 1999 and 2018 and to discuss the implications for disease surveillance in the region.

## 2. Material and Methods

### 2.1. Study Population

**Figure 1:**
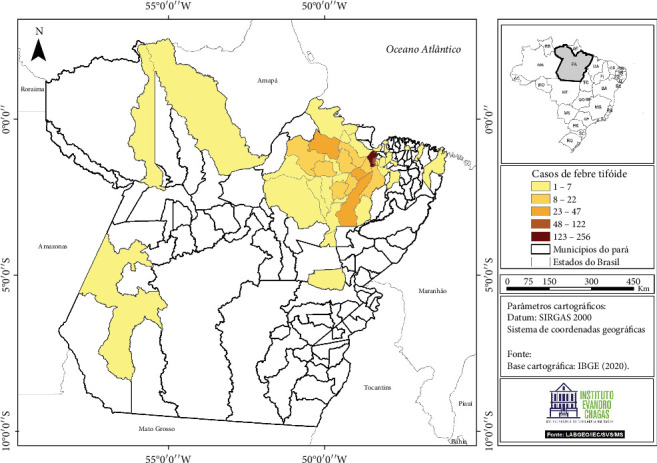
Map of the state of Pará showing the 43 municipalities included in this study.

This study analyzed clinical records of patients (children, adults, and elderly individuals) from 43 municipalities in the state of Pará, Brazil (Figure 1). Data were collected from clinical–epidemiological records to obtain information on sociodemographic and clinical–epidemiological characteristics.

### 2.2. Sample Selection

Only confirmed cases of TF diagnosed at the IEC between 1999 and 2018 were included. Confirmatory diagnosis was established through blood culture, stool culture, or polymerase chain reaction (PCR).

### 2.3. Bacterial DNA Extraction, Gene Targets, and Primers

Bacterial DNA was extracted using the DNA IQ Kit (Promega), following the manufacturer's instructions. PCR amplification targeted the *viaB*, *prt*, *fliC-d*, and *invA* gene regions using primers as described by Levy et al. [16] and Kumar et al. [17]. Each PCR reaction had a final volume of 25 *μ*L, containing 20 ng of DNA, 10 mM Tris-HCl (pH 8.5), 50 mM KCl, 1.5 mM MgCl_2_, 1.25 mM of each dNTP, 1.25 mM of each primer, and 0.5 units of Platinum Taq DNA Polymerase (Invitrogen). Thermocycling conditions were as follows: initial denaturation at 95°C for 4 min, followed by 35 cycles of denaturation at 95°C for 1 min, annealing at 55°C for 1 min, and extension at 72°C for 1 min.

### 2.4. Specificity of PCR Assay

To assess the specificity of the primers, amplification reactions were performed using DNA from various gastrointestinal microorganisms and pathogens, including *Escherichia coli*, *Salmonella* Paratyphi A, *Salmonella* Typhimurium, *Salmonella* Panama, *Proteus mirabilis*, and *Shigella flexneri*.

### 2.5. Study Variables

The variables used for statistical analysis included diagnostic method, date of sample collection, age, sex, education level, occupation, municipality of residence, area of residence (urban/rural), type of housing, type of terrain, water source, sewage disposal method, garbage disposal method, history of contact with suspected cases, number of cohabitants, presence of other positive individuals in the household, hospitalization, prior antibiotic use, duration, and type of symptoms.

### 2.6. Statistical Analysis

All data were entered into a Microsoft Excel 2016 database and exported to the Statistical Package for the Social Sciences (SPSS), Version 26. Descriptive statistical analysis was conducted, with absolute and relative frequencies calculated for categorical variables. Results were presented in tables and graphs.

The chi-square test (*χ*^2^) was used to assess differences in proportions and the distribution of categorical variables. A significance level of *α* ≤ 0.05 was adopted. For multivariate analysis, variables with a *p* value < 0.05 in the chi-square test were further analyzed using multiple logistic regression.

### 2.7. Ethical Considerations

This study was approved by the Research Ethics Committee of the IEC (CEP/IEC/SVS/MS) under Number 30482220.7.0000.0019, issued on July 3, 2020.

## 3. Results

### 3.1. Study Population

This study analyzed clinical records of patients (children, adults, and elderly individuals) from 43 municipalities in the state of Pará, Brazil (Figure 1). Data were collected from clinical–epidemiological records to obtain information on sociodemographic and clinical–epidemiological characteristics.

A total of 683 clinical–epidemiological records were analyzed during the study period. The distribution of cases by sex revealed a predominance of males over females. The age range of affected individuals varied from 1 to 88 years, with the majority of cases occurring in adults aged 20–59 years.

**Figure 2:**
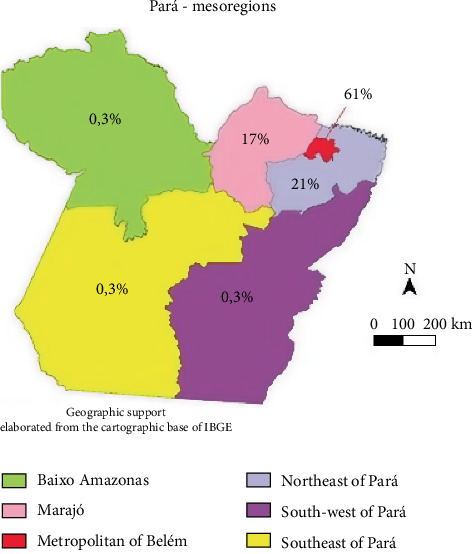
Geographic distribution of typhoid fever (TF) cases by city of residence, organized by mesoregions in the state of Pará, from 1999 to 2018.

With regard to education level, most individuals had low educational attainment.  Table 1 presents the baseline sociodemographic data of patients diagnosed with TF.  Figure 2 shows the geographical distribution of cases according to the patients' municipalities of residence, grouped by mesoregions in the state of Pará and served by the IEC.

Statistical analysis of the data revealed significant associations between sociodemographic variables—such as sex, age group, education level, and occupation—and the occurrence of TF. The higher proportion of cases among males, adults, individuals with lower education levels, and students suggests that these groups may be more vulnerable to infection or more exposed to risk factors.

In summary, Table 1 provides valuable insights into the sociodemographic profile of TF cases, highlighting population groups with a higher prevalence of the disease. Although these differences were statistically significant, the presence of missing data in some variables warrants caution in their interpretation.

Figure 2 also demonstrates a strong concentration of TF cases in the Metropolitan Region of Belém, followed by the Northeast Pará and Marajó mesoregions. The remaining mesoregions had very few cases. This uneven geographic distribution suggests the influence of regional-specific factors and underscores the need for targeted public health interventions.

### 3.2. Exposure Variables

The majority of patients resided in urban areas. Most lived in masonry homes situated on dry land without a history of flooding.

As for water supply, the majority of cases reported using water from the public supply system. Regarding sewage and solid waste disposal, most households relied on septic tanks and public waste collection services.

Concerning the likely source of infection, most patients reported the habitual or occasional consumption of *açaí*, followed by the ingestion of unspecified suspicious foods shortly before the onset of symptoms.

The number of cohabitants per household varied, but most patients reported living with two to six other individuals. Additionally, most cases indicated that at least one other family member had a suspected or confirmed case of TF.

It is important to note that more than half of the records lacked complete information for several exposure-related variables, including type of housing, land characteristics, water source, sewage and waste disposal, household size, and presence of other infected individuals. These omissions were marked as “uninformed” in the dataset.  Table 2 presents the available data on housing conditions.

Despite the data limitations, statistical analysis indicated significant associations between housing conditions, potential exposure factors, and the occurrence of TF. However, the high proportion of missing information significantly limits the ability to draw definitive conclusions about the specific environmental or structural factors associated with increased TF risk.

A substantial proportion of cases (> 50%) had missing data for key variables, including housing type, water source, and number of cohabitants. This limitation may compromise the internal validity of the multivariable analysis and introduce bias in the estimates, potentially affecting the accurate identification of factors associated with the outcomes of interest.

In the multivariate logistic regression analysis, several variables were significantly associated with TF. Urban residence was linked to higher odds of infection (OR = 1.85; 95% CI: 1.20–2.85; *p* = 0.005), as was residence in wooden housing (OR = 1.62; 95% CI: 1.01–2.61; *p* = 0.045) and living on flood-prone terrain (OR = 2.45; 95% CI: 1.34–4.47; *p* = 0.003).

With respect to environmental exposures, the use of artesian well water (OR = 2.12; 95% CI: 1.18–3.79; *p* = 0.011) and river water (OR = 3.01; 95% CI: 1.45–6.25; *p* = 0.002) were both associated with elevated risk. Inadequate waste disposal practices, specifically the use of latrines, also emerged as a significant risk factor (OR = 2.78; 95% CI: 1.11–6.95; *p* = 0.029), whereas access to public garbage collection demonstrated a protective effect (OR = 0.64; 95% CI: 0.43–0.96; *p* = 0.030).

Behavioral factors contributed to disease risk as well. Consumption of açaí (OR = 2.33; 95% CI: 1.39–3.91; *p* = 0.001) and ingestion of suspicious food items (OR = 1.97; 95% CI: 1.18–3.30; *p* = 0.009) were both significantly associated with increased odds of infection.

Household characteristics also played a notable role. Living with 7–11 cohabitants nearly doubled the odds of infection (OR = 1.88; 95% CI: 1.03–3.44; *p* = 0.038), while having at least one additional infected household member nearly tripled the risk (OR = 2.95; 95% CI: 1.74–5.00; *p* < 0.001), underscoring the potential for intrahousehold transmission.

Overall, these findings highlight the multifactorial nature of TF transmission, shaped by structural, environmental, and behavioral determinants. They underscore the urgent need for integrated interventions aimed at improving water access, strengthening sanitation infrastructure, and promoting safe food practices.

### 3.3. Clinical Variables

**Figure 3:**
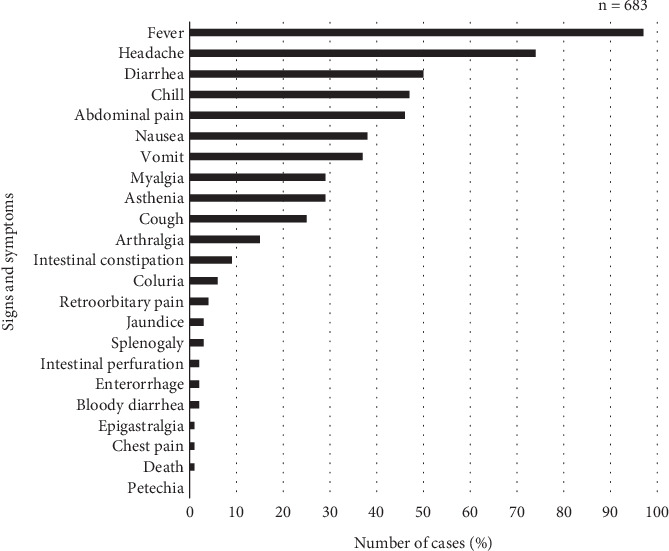
Clinical characteristics of TF positive individuals.

Table 3 and Figure 3 present the clinical characteristics of patients diagnosed with TF, as recorded in the clinical–epidemiological database. With regard to hospitalization, most patients did not require inpatient care and were treated on an outpatient basis. Similarly, the majority reported not having used antibiotics prior to seeking medical attention. The duration of symptoms ranged from 1 to more than 52 days, with the majority of cases falling within the 12- to 21-day range.

Among the patients, 42.6% reported not using antibiotics before diagnosis, whereas 26.2% reported prior use of antibiotics despite the absence of laboratory confirmation. This practice of improper or self-medication may reflect diagnostic challenges in distinguishing TF from other febrile illnesses, as well as the ease of access to antibiotics without a prescription, particularly in peripheral regions of the state.

Statistical analysis showed significant associations between hospitalization status, antibiotic use, and symptom duration with TF occurrence. The highly significant *p* value related to symptom duration suggests a strong association between the time from symptom onset to medical evaluation and the likelihood of confirmed TF diagnosis. The concentration of cases within the 12–21-day range may provide insights into the disease's typical clinical course and diagnostic window.

Regarding symptomatology, the most frequently reported symptoms were fever (660 cases), headache (507), diarrhea (344), chills (323), abdominal pain (314), nausea (258), and vomiting (249). Severe complications such as intestinal perforation and death were rare, occurring in less than 1% of cases (Figure 3).

Overall, the graph clearly demonstrates the hierarchy of signs and symptoms in the analyzed TF cases, with fever being the predominant symptom, followed by headache and diarrhea. The low frequency of severe signs suggests a generally nonfatal progression in the studied sample.

### 3.4. Diagnostic Methods

As shown in Table 4, blood culture and stool culture were the most commonly employed diagnostic methods for TF, accounting for 50.8% (*n* = 347) and 38.0% (*n* = 257) of the cases, respectively. Among these, 5.6% (*n* = 34) of cases were diagnosed using both methods, with positive results recorded in the official reporting forms.

Since its implementation in 2013, PCR has been used in 16.5% (*n* = 113) of cases. Notably, in 2014 and 2015, the number of diagnoses made by PCR surpassed those made by conventional culture techniques. However, in subsequent years, the number of PCR tests decreased, primarily due to shortages of necessary reagents and supplies.

Table 4 illustrates the evolution of diagnostic practices for TF over the past two decades. In the earlier years of the series, traditional culture-based methods predominated. From 2013 onward, PCR gradually gained relevance, reflecting both technological advancements and increased access to molecular diagnostic tools. The observed year-to-year variation in the application of diagnostic methods, as well as the low frequency of copositivity among tests, highlights the need for ongoing methodological improvements and greater consistency in disease surveillance.

### 3.5. Annual and Monthly Distribution of TF

Table 5 presents the total population of the state of Pará alongside the cases of TF identified by the IEC and reported to the Notifiable Diseases Information System (SINAN) over a 20-year period. No uniform pattern of annual case distribution was observed. The number of cases varied significantly, ranging from a minimum of 16 in 2000 to a peak of 95 cases in 2014.

A notable discrepancy occurred in 2010, when the IEC confirmed 39 cases, but only 23 were reported to SINAN, indicating underreporting of 16 cases. This gap highlights the importance of strengthening notification systems to ensure more accurate epidemiological surveillance. The variations in annual case numbers call for further investigation to identify possible outbreak events, environmental determinants, or changes in public health infrastructure and practices.

**Figure 4:**
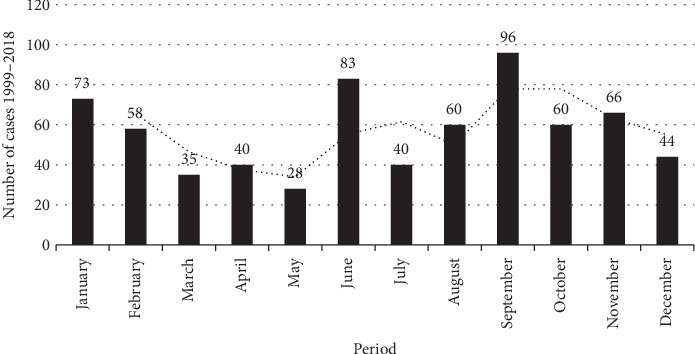
Monthly distribution of the number of cases reported by the IEC in the state of Pará, from 1999 to 2018.

The monthly distribution revealed a seasonal pattern, with the majority of cases (59.3%) occurring between June and November—coinciding with the Amazonian dry season. Additional increases were noted in January (10.7%) and February (8.5%) (Figure 4). Despite slight monthly fluctuations, no clear upward trend in incidence was detected across the entire series. Nonetheless, the seasonal concentration of cases supports literature findings that associate TF with drier months, when reduced rainfall may increase risk factors related to hygiene and water contamination.

## 4. Discussion

The highest frequency of TF cases was recorded among male patients, accounting for 65.3% of all cases. This gender distribution is consistent with findings reported by Maravilla [20], who observed a 61% incidence among males in an epidemiological study conducted in a hospital in El Salvador. The predominant age group in the present study comprised individuals aged 20–59 years, representing 58.1% of cases, with a mean age of 33 years. These findings align with data from the Brazilian Epidemiological Bulletin on Neglected Tropical Diseases [21], which reported the highest prevalence among individuals aged 20–34 years (28.75%) among confirmed cases.

Analysis of exposure-related variables revealed that most patients resided in urban areas (81.4%), lived in masonry houses (22.1%) with septic tanks (28.3%), on dry land (21.2%), and were supplied by the public water system (23.9%). Additionally, 35.1% had access to regular public waste collection, 24.2% lived with two to six cohabitants, and 35% reported at least one suspected or confirmed TF case in the same household.

According to the Epidemiological Bulletin on Neglected Tropical Diseases [21], urban residents in the Northern Region of Brazil were the most affected (81.54%). The most frequently reported sources of exposure included the consumption of untreated water (25.02%) and contaminated food (14.02%), corroborating the findings of this study.

The Trata Brasil Institute's annual Basic Sanitation Ranking for the 100 largest Brazilian cities, based on 2019 data, ranked Belém 96th in total water supply coverage (71.5%) and Ananindeua 100th (32.5%). Regarding sewage treatment coverage, Belém ranked 95th (15.77%) and Ananindeua 99th (2.08%), both substantially below the national averages of 93.51% and 74.47%, respectively [22].

Hospitalization was required in 15.2% of the cases analyzed. According to the Ministry of Health's Hospital Information System (SIH), 2145 hospitalizations due to typhoid and paratyphoid fever occurred in Pará between 1999 and 2018 [23]. The most frequently reported clinical symptoms were fever, headache, diarrhea, chills, abdominal pain, nausea, vomiting, asthenia, and myalgia. Fewer than 1% of patients progressed to intestinal perforation and/or death, with such outcomes recorded in the years 2005, 2008, 2014, and 2015. These figures are within the global complication rate of approximately 3% for TF [24].

Geographically, the highest number of cases was reported in the metropolitan region of Belém, accounting for 61% of the total, with the municipalities of Belém (37.2%) and Ananindeua (17.9%) being the most affected. Despite having relatively high Human Development Index (HDI) scores—0.746 and 0.718, respectively—these municipalities still exhibit considerable deficiencies in basic sanitation infrastructure. Table 5 shows that, over the 20-year period analyzed, the number of reported cases declined annually despite population growth, possibly due to improvements in sanitation, albeit limited, and the historical use of nonspecific diagnostic methods such as the Widal test until mid-2013 [25–27].

Zorgani and Ziglam [28] noted that the Widal test has a sensitivity as low as 63%, which is inferior to that of blood culture and PCR, both of which exceed 95% sensitivity. Brazil and Turkey have officially reported that the Widal test is unreliable for differential diagnosis, as *Salmonella* Typhi shares O and H antigens with other serotypes and enterobacteria, resulting in false positives. Moreover, some patients with positive blood cultures may not produce sufficient antibodies detectable by agglutination tests, leading to false negatives [29].

In 2010, 16 cases were underreported when comparing records from the IEC and the SINAN. Since 2013, the IEC has been officially designated as a notifying unit in the state of Pará. Previously, the institute referred cases to local health units for notification, which often led to data loss and compromised the implementation of appropriate epidemiological control measures [30].

Finally, underreporting of TF remains a major obstacle for epidemiological surveillance. It undermines accurate assessment of the disease burden and impedes the formulation of effective public health policies. The discrepancies observed between data reported by the IEC and the SINAN point not only to operational deficiencies in case reporting but also to broader systemic challenges in surveillance capacity.

Notable peaks in disease incidence occurred in the years 1999, 2001, 2004, 2014, and 2015, corresponding to outbreak events in the municipalities of Mojú (1999), Anajás (2001 and 2004), Castanheira (Belém metropolitan region, 2014), and Breves (2015) [31].

Blood and stool cultures were the primary diagnostic methods, with blood culture demonstrating superior sensitivity. Only 5.6% of cases tested positive with both methods, indicating limited copositivity. The predominance of blood culture as a diagnostic tool significantly influences case confirmation and reporting in Ananindeua and surrounding areas, particularly in the acute phase of the disease.

According to Brusch et al. [32], blood and stool cultures yield positive results in 85%–90% of cases during the first weeks of infection, but their sensitivity declines to 20%–30% in later stages. The implementation of PCR, especially from 2014 onward, played a key role in identifying suspected cases with negative cultures, often due to prior antibiotic use. This was evident during the outbreaks in Belém and Breves [31].

Although prior use of antibiotics was recognized as a variable of interest, this study did not perform a stratified analysis to quantify its direct impact on diagnostic yield. The absence of this approach limits a more detailed understanding of how prior antibiotic exposure affects the sensitivity of the diagnostic methods used, particularly in a context where diagnostic practices varied over time.

Although not yet well characterized, TF appears to follow a seasonal pattern, with a higher incidence during the drier months. The state of Pará is divided into seven major hydrographic regions and experiences a humid equatorial climate with an annual mean temperature of 25.6°C and average precipitation of 2157 mm. Two distinct seasons prevail: the rainy season (December–May) and the drier season (June–November) [33].

Regarding probable sources of exposure, 21% of patients reported consuming açaí prior to symptom onset, followed by other suspected food items (20%). Açaí, a native fruit widely consumed in Pará, is harvested predominantly in the second half of the year and plays a significant role in the regional diet and economy [34, 35].

Studies by Santos [34] identified microbial contamination in both the production and storage phases of açaí, including *E. coli*, fecal coliforms, molds, and yeasts in frozen pulp. Factors contributing to contamination include temperature, humidity, poor hygiene of handlers and equipment, and the use of untreated water during processing [35].

A high percentage of missing data on exposure variables was noted in this study, which limits accurate assessment of the disease burden and hinders the development of effective preventive strategies by epidemiological surveillance services.

Despite being the state capital, the Belém metropolitan area still experiences considerable deficiencies in sanitation and access to potable water. Such precarious conditions are directly linked to higher disease incidence, highlighting the relationship between TF and environmental factors such as sanitation and hygiene, particularly in food handling. Investments in clean water, sanitation, and hygiene (WASH) infrastructure are essential to prevent transmission.

TF remains a significant yet underrecognized global public health concern, especially in marginalized populations lacking access to diagnostic tools and surveillance systems. The overuse of antibiotics based on clinical suspicion has contributed to the emergence of MDR strains, further complicating disease control [14, 15].

## 5. Conclusion

This 20-year retrospective study offers novel insights into the epidemiological and clinical profile of TF in the state of Pará, contributing valuable data to public health surveillance efforts. The persistence of the disease is closely linked to sociodemographic conditions, population mobility in endemic areas, and failures in food handling practices. These findings underscore the urgent need for increased investment in research on TF, particularly in endemic regions, to support evidence-based decision-making and inform targeted interventions. Furthermore, the results highlight the importance of addressing the broader context of social vulnerability in affected communities through integrated public health and infrastructure policies.

## Figures and Tables

**Table 1:** Distribution of TF cases according to sex, age group, schooling, marital status, and place of origin.

**Variables**	**Total**	**Frequency (%)**	**χ** ^2^ **adherence**	**p**
Sex				
Male	446	65.3	63.9	< 0.0001
Female	237	34.7	
Total	683	100		
Age group				
Children (1–12)	127	18.6	852.2	< 0.0001
Young (13–19)	139	20.3	
Adults (20–59)	397	58.1	
Senior (60–88)	18	2.7	
Uninformed	2	0.3	
Total	683	100		
Schooling				
Nonliterate	25	3.7	580.1	< 0.0001
Literate	16	2.3	
Elementary school	298	43.6	
High school	209	30.6	
Higher education	42	6.1	
Uninformed	93	13.6		
Total	683	100		
Occupation				
Autonomous	89	13.0	355.6	< 0.0001
Employee	111	16.3	
Unemployed	34	5.0	
Kindergarten	21	3.1	
Student	263	38.5	
Uninformed	165	24.2	
Total	683	100		

**Table 2:** Distribution of TF cases according to housing conditions.

**Variables**	**Total**	**Frequency (%)**	**χ** ^2^ **adherence**	**p**
Area of residence				
Urban	556	81.4	718.1	< 0.0001
Rural	95	13.9	
Uninformed	32	4.7	
Total	683	100		
Type of building				
Masonry	151	22.1	387.5	< 0.0001
Wood	149	21.8	
Mixed	12	1.8	
Uninformed	371	54.3	
Total	683	100		
Type of land				
Dry	145	21.2	723.8	< 0.0001
Flooded	61	8.9	
Mixed	13	1.9	
Uninformed	464	67.9	
Total	683	100		
Source of water				
Mineral water	6	0.9	784.2	< 0.0001
Public system	163	23.9	
Artesian well	66	9.7	
Open pit	46	6.7	
River	38	5.6	
Uninformed	364	53.3	
Total	683	100		
Destination waste				
Latrine	19	2.8	1.4	< 0.0001
Biological cesspool	193	28.3	
Septic tank	36	5.3	
Hole	58	8.5	
River	6	0.9	
Uninformed	371	54.3	
Total	683	100		
Garbage disposal				
Public collection	240	35.1	794.7	< 0.0001
Wasteland	26	3.8	
Burned	38	5.6	
Buried	2	0.3	
Uninformed	377	55.2	
Total	683	100		
Contact history			765.5	< 0.0001
Untreated water	12	1.7		
Suspicious food	137	20.0		
Açaí consumption	143	21.0		
Residence	61	9.0		
Trip	75	11.0		
Uninformed	255	37.3		
Total	683			
Cohabitants				
1	5	0.7	415.9	< 0.0001
2–6	165	24.2	
7–11	59	8.6	
12–16	6	0.9	
17+	1	0.1	
Uninformed	447	65.4	
Total	683	100		
Other positive residents				
1	239	35.0	591.7	< 0.0001
2–3	21	3.1	
4–5	6	0.9	
6+	2	0.3	
Uninformed	415	60.8	
Total	683	100		

**Table 3:** Distribution of TF cases according to hospitalization, previous antibiotic use, and days of symptoms.

**Variables**	**Total**	**Frequency (%)**	**χ** ^2^ **adherence**	**p**
Hospitalization				
No	468	68.5	380.665	< 0.0001
Yes	104	15.2	
Uninformed	111	16.3	
Total	683	100.0		
Use of antibiotic				
No	291	42.6	28.966	< 0.0001
Yes	179	26.2	
Uninformed	213	31.2	
Total	683	100.0		
Symptom time			814.116	< 0.0001
1	3	0.4		
2–11	182	26.6		
12–21	295	43.2		
22–31	126	18.4		
32–41	31	4.5		
42–51	12	1.8		
52+	5	0.7		
Uninformed	29	4.2		
Total	683	100.0		

**Table 4:** Distribution of diagnostic methods according to the year they were performed.

**Year**	**Hemoculture**	**Coproculture**	**Hemo and Copro**	**PCR**
	**N**	**N**	**N**	**N**
1999	51	21	6	—
2000	11	7	2	—
2001	24	19	3	—
2002	6	21	1	—
2003	7	10	—	—
2004	22	33	5	—
2005	10	18	2	—
2006	11	18	3	—
2007	11	16	—	—
2008	16	19	—	—
2009	18	3	—	—
2010	26	14	1	—
2011	16	7	2	—
2012	22	12	1	—
2013	11	5	—	7
2014	28	13	2	56
2015	15	2	1	30
2016	17	6	2	9
2017	19	5	2	3
2018	6	8	1	8
Total	347	257	34	113

**Table 5:** Historical series of the population of the state of Pará compared to TF cases reported by IEC and SINAN, from 1999 to 2018.

**Year**	**Population**	**No. of cases IEC**	**No. of cases SINAN**
1999	5,886,463	66	⁣^∗^
2000	6,192,307	16	⁣^∗^
2001	6,341,711	40	27
2002	6,453,699	26	103
2003	6,574,990	17	209
2004	6,695,940	50	141
2005	6,970,591	27	107
2006	7,110,462	25	75
2007	7,249,184	27	74
2008	7,321,493	35	57
2009	7,431,041	21	71
2010	7,581,051	39	23
2011	7,688,593	21	21
2012	7,821,276	33	53
2013	7,969,654	23	35
2014	8,073,924	95	112
2015	8,175,113	46	57
2016	8,272,724	30	35
2017	8,366,628	25	38
2018	8,513,497	21	26

*Note:* Asterisks denote no data, system active from 2001. *Source:* IBGE [18]; DATASUS [19].

## Data Availability

The data that support the findings of this study are available on request from the corresponding author. The data are not publicly available due to privacy or ethical restrictions.
